# The value of 18F-FDG PET/CT combined with 3D quantitative technology and clinicopathological features in predicting prognosis of NSCLC

**DOI:** 10.3389/fonc.2025.1533569

**Published:** 2025-04-08

**Authors:** Yuling Su, Siwen Qiu, Jinyu Wang

**Affiliations:** Department of Nuclear Medicine, Zhuhai People’s Hospital (The Affiliated Hospital of Beijing Institute of Technology, Zhuhai Clinical Medical College of Jinan University), Zhuhai, China

**Keywords:** lung cancer, PET/CT, prognosis, 3D quantitative technology, NSCLC

## Abstract

**Objective:**

To investigate the value of Fluorine-18 Fluorodeoxyglucose (^18^F-FDG) Positron Emission Tomography/Computed Tomography (PET/CT) combined with 3D quantitative technology and clinicopathological features in predicting the prognosis of non-small cell lung cancer (NSCLC).

**Methods:**

A retrospective review was performed for patients who underwent PET/CT and curative resection of NSCLC between January 2016 and June 2019 in our hospital. PET/CT data, clinical features, and pathology results were collected. Gross tumor volume (GTV) was delineated on CT images by ITK-SNAP software. The prognosis was followed up, and the study endpoint was progression-free survival (PFS). Receiver operating characteristic curve (ROC) was used to initially assess the relationship between each parameter and PFS, and parameters were grouped accordingly. Cox proportional hazards regression was used to develop models based on clinicopathological features to predict prognosis of NSCLC patients. Kaplan–Meier method was used to draw the survival curves.

**Results:**

A total of 128 patients were enrolled in the study with PFS of 8–96 months. Univariate analysis demonstrated that age, SUVindex (the ratio of SUVmax of lesion to SUVmax of liver), metabolic tumor volume (MTV), Dmax (the largest diameter), GTV, lymph node metastasis (LNM), and TNM staging are significantly related to recurrence (all p<0.05). The multivariate analysis showed that only age, SUVindex, and LNM were independent prognostic factor for PFS (all p < 0.05).

**Conclusions:**

Although 18F-FDG PET/CT combined with 3D quantitative technique were helpful in predicting PFS in NSCLC, only age, SUVindex, and LNM were independent predictors for PFS.

## Introduction

Lung cancer is the leading cause of cancer-related deaths worldwide with a 5-year survival rate of approximately 15% (1, 2), of which NSCLC is the most common type, accounting for more than 85% (3). With the continuous development of early diagnosis and adjuvant therapy in recent years, the prognosis of NSCLC has been improved, but the recurrence rate after treatment is still high. Although many studies have been conducted to find factors that could predict the prognosis, the best prognostic predictors available are still debated (4–6).

PET/CT has been used in lung cancer for more than 20 years. Many studies have confirmed that PET/CT plays a crucial role in the staging, efficacy evaluation, and detection of recurrence in NSCLC (7, 8). The maximum standard uptake value (SUVmax) is the most commonly used PET/CT parameter, but it is susceptible to a variety of factors and measured by different scanners varies widely. A study has shown that SUVindex is a simple and reliable predictor of prognosis in early-stage lung cancer (9). MTV represents the metabolic information of the whole tumor and can provide both metabolic activity and volume information. One study showed that MTV was significantly better than SUVmax in predicting lung cancer prognosis (10), but the threshold value used to measure MTV is still debated (11).

3D quantitative technology based on genomics has been a hot research topic in recent years, and ITK-SNAP software is commonly used in genomics at home and abroad to accurately extract 3D information of lesions (12). Previous studies have found that the assessment of intra-tumor features on CT is useful for predicting overall survival (OS) and PFS in NSCLC (13, 14).

In recent years, a study has also shown that systemic inflammation and nutritional status are closely related to malignant tumorigenesis, progression, and prognosis (15). Many inflammatory and nutritional markers based on serological indicators have been applied to predict the prognosis of NSCLC (16–18).

Therefore, we evaluated the value of PET/CT combined with 3D quantitative technique and clinicopathological features in predicting postoperative recurrence in NSCLC patients to look for new indicators that can accurately estimate the prognosis.

## Materials and methods

### Study design

All enrolled cases were NSCLC patients who underwent FDG PET/CT and radical resection within 2 weeks in our hospital from January 2016 to June 2019. A total of 68 patients underwent postoperative chemotherapy or targeted therapy. Pathological classification was performed according to the 2021 WHO classification of thoracic tumors and TNM staging according to the American Joint Committee on Cancer (AJCC) eighth edition. This study was approved by the Institutional Review Board of Zhuhai People’s Hospital in Guangdong Province, China. The requirement for informed consent was waived due to the retrospective nature of the study design.

### Inclusion and exclusion criteria

Inclusion criteria were as follows: (I) complete data were available; (II) patients did not undergo any anti-tumor therapy or had no history of malignancy before PET/CT examination; (III) all patients underwent curative resection and confirmed to be NSCLC; and (IV) all patients underwent standardized postoperative treatment.

Exclusion criteria were as follows: (I) poor image quality or undistinguished lesion boundary or (II) without standardize treatment or without review after surgery.

### Imaging equipment and methods

The Philips Gemini TF PET/CT scanner (Philips Medical Systems, Netherlands) was applied to conduct the PET/CT imaging. Patients fasted for more than 4 h, and fasting blood glucose was measured before intravenous injection of 18F-FDG (Atom High-tech Isotope Pharmaceutical Co., Ltd., Guangzhou, China; radiochemical purity > 95%, 4.07–5.18 MBq/kg). Patients were allowed to rest for 60 min in a quiet, warm, dark condition before PET/CT examination.

First low-dose CT scan was performed for attenuation correction and anatomical location with a tube voltage of 80 kV, a tube current of 150 mAs, and a layer thickness of 2 mm. PET acquisition was performed in three-dimensional mode with the scope of acquisition from the upper thigh to the cranial roof, and the scanning time was 70 s/bed.

### Image analysis and segmentation

The EBW system (Philips, Netherlands) was used for image processing. The following parameters including MTV of primary foci with the threshold of 40%SUVmax, Dmax (the largest diameter of primary foci), SUVmax of the primary foci, and normal liver parenchyma were measured. SUVindex = SUVmax of the primary foci/SUVmax of normal liver parenchyma (19).

ITK-SNAP (Version 3.8) was used for image segmentation. Two experienced nuclear physicians outlined the CT axial images layer by layer until the entire primary lesion was outlined, and the GTV was automatically calculated by the software. When the boundary was not clear, the PET images were referred to. The internal tumor cavity was avoided while outlining. The average of the measurements were recorded.

### Follow-up

Patients were followed up at 3-month intervals for the first 3 years after the operation, at 6-month intervals for the next 2 years, and annually thereafter. Patients were followed up via the HIS system and telephone. The beginning time was the date of surgery, and the termination was tumor recurrence or deadline of 31 December 2023. The recurrence was confirmed by imaging or pathology. PFS was defined as the time from initial treatment to tumor progression, death, or deadline.

### Statistical analysis

GraphPad Prism (Version 9.2), R Language, and SPSS (Version 25.0, IBM) were used for data analysis. Normally distributed data were expressed as mean ± standard deviation, and non-normal data were expressed as the median (interquartile range). Comparisons between two groups of normally distributed data were analyzed using a Student’s t-tests, and comparisons between groups of non-normal data were analyzed using the Mann–Whitney U test. The prognostic value of each parameter for PFS was initially assessed using the receiver operating characteristic (ROC) curve. Univariate and multivariate analyses were performed with the COX proportional risk regression model to obtain the independent predictors of PFS. Survival curves were plotted using the Kaplan–Meier method, and the log-rank test was used to analyze the survival differences between groups. A p-value<0.05 was considered statistically significant.

## Results

In total, 128 patients were analyzed in the present study, including 79 cases in stage I, 18 cases in stage II, and 31 cases in stage III. A total of 34 patients experienced recurrence with an overall median PFS of 48 months (**Table 1**), and three patients experienced new cancers beyond 5 years.

**Table 1:** Univariate analysis of PFS (Cox proportional hazard model).

Variables	HR (95%CI)	P-value
Age (years) ≤60 (n=48) >60 (n=80)	2.161(1.003–4.655)	0.049
Gender Male (n=74) Female (n=54)	1.019 (0.516–2.011)	0.957
SUVindex ≤2.36 (n=80) >2.36(n=48)	3.541 (1.749–7.170)	<0.01
MTV ≤21.18 (n=107) >21.18 (n=21)	2.433 (1.178–5.028)	0.016
GTV (cm^3^) ≤26.1 (n=107) >26.1 (n=21)	2.256 (1.091–4.666)	0.028
Pathological type Adenocarcinoma (n=102) Else (n=26)	0.505 (0.177–1.143)	0.200
lymphocyte count Low (n=5) Normal (n=123)	1.914 (0.453–8.084)	0.377
Serum albumin level Low (n=38) Normal (n=90)	1.802 (0.903–3.595)	0.095
LNM No (n=86) Yed (n=42)	4.057 (2.002–8.223)	<0.01
Stage I+II (n=97) III (n=31)	3.658 (1.793–7.461)	<0.01
Dmax (mm) ≤41.5 (n=110) >41.5 (n=18)	2.288 (1.085–4.826)	0.030
Adjuvant therapy No Yes	1.620 (0.750–3.498)	0.219

PFS, progression-free survival; MTV, metabolic tumor volume; GTV, gross tumor volume; LNM, lymph node metastasis; Dmax the largest diameter.

### Univariate analysis of progression-free survival

Univariate analysis revealed that the age, SUVindex, MTV, GTV, LNM, stage, and Dmax were correlated with PFS (all p <0.05). The hazard ratio (HR) was 2.161 (1.003–4.655), 3.541 (1.749–7.170), 2.433 (1.178–5.028), 2.256 (1.091–4.666), 4.057 (2.002–8.223), 3.658 (1.793–7.461), 2.288 (1.085–4.826), respectively. The gender, pathological type, and adjuvant therapy were not correlated with PFS (all p> 0.05) (**Table 1**).

### Multivariate analysis with respect to PFS and survival curves

Combinatorial effects and interactions between Age, SUVindex, MTV, GTV, Stage, LNM, and Dmax were examined using Cox proportional hazard models. It was found that only Age, SUVindex, and LNM remained independent predictors of PFS (all p < 0.05), with HR of 3.456 (1.483–8.053), 3.606 (1.510–8.612), and 3.123 (1.067–9.139), respectively (**Table 2**).

**Table 2:** Results of multivariate analysis of DFS (Cox proportional hazard model).

Variables	HR (95%CI)	P-value
Age (years)	3.456 (1.483–8.053)	0.004
SUVindex	3.606 (1.510–8.612)	0.004
MTV	1.307 (0.205–8.354)	0.777
GTV	1.259 (0.145–10.950)	0.834
Stage	1.227 (0.420–3.580)	0.708
LNM	3.123 (1.067–9.139)	0.038
Dmax	0.397 (0.044–3.594)	0.411

PFS, progression-free survival; MTV, metabolic tumor volume; GTV, gross tumor volume; LNM, lymph node metastasis; Dmax, the largest diameter.

**Figure 1:**
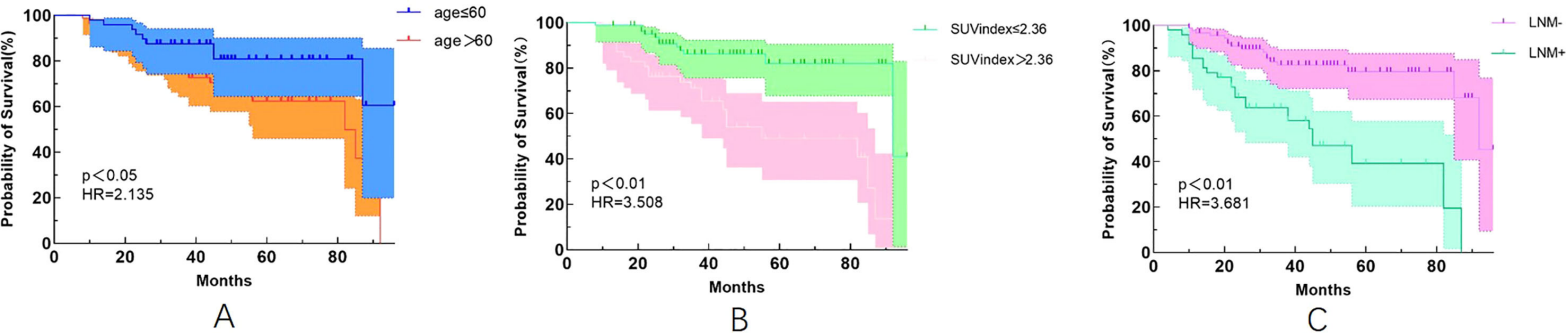
Kaplan-Meier survival curves for different variables. **(A)** Age (p<0.05); **(B)** SUVindex (p<0.05); **(C)** LNM (p<0.05).

Our study also found that patients with age >60 years were 2.135 times more likely to experience recurrence than those with age ≤60 years (**Figure 1A**). Patients with an SUVindex of >2.36 were 3.508 times more likely to experience recurrence than those with a SUVindex of ≤2.36 (**Figure 1B**). Patients with LNM were 3.681 times more likely to experience recurrence than those without LNM (**Figure 1C**).

### Survival ROC curves parameters for predicting PFS

**Figure 2:**
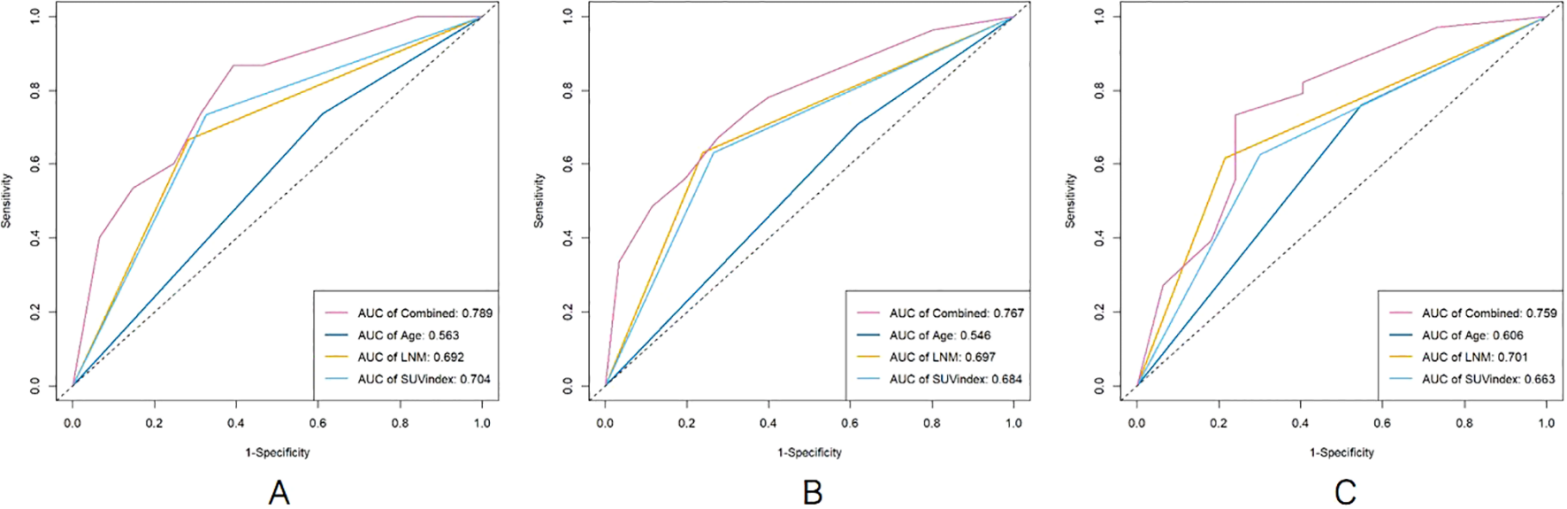
The comparison of survival ROC curves for predicting PFS [**(A)**, 2 years; **(B)**, 4 years; **(C)**, 6 years].

The survival ROC curve analysis for different time points of Age, SUVindex, LNM, and the Combined showed that the AUC value of the Combined was higher than the other parameters (**Figure 2**).

### Relationship between different stages and recurrence

**Figure 3:**
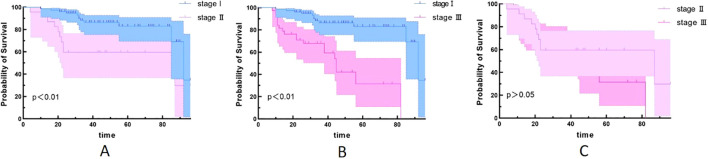
Kaplan–Meier survival curves for different stages. **(A)** stage I and stage II (p<0.05); **(B)** stage I and stage III (p<0.05); **(C)** stage II and stage III (p>0.05).

Since univariate analysis showed that staging was associated with PFS, we further compared survival outcomes across different stages. We found that stage III patients are more prone to experience recurrence compared to stage I and II patients (**Figures 3A, B**). However, the difference in survival between stage II and stage III patients was not statistically significant (**Figure 3**).

## Discussion

This study explored the value of 18F-FDG PET/CT combined with 3D quantitative technique and clinicopathological features in predicting the postoperative risk of recurrence in NSCLC, in order to identify new reliable and sensitive prognostic indicators, stratify patients according to prognosis, and thus develop appropriate treatment strategies.

In this study, univariate analysis indicated that Age, MTV, GTV, SUVindex, LNM, and Stage were predictive factors of tumor recurrence, and patients were divided into two prognostic subgroups. In the multivariate analysis, Age, SUVindex, and LNM were found to be independent predictors of disease recurrence, thus establishing a model to predict PFS of NSCLC.

Previous studies (20–22) have shown that the prognosis of lung cancer is related to many factors, such as age, clinical stage, cell type, treatment modality, primary tumor size, and SUVmax, but the conclusions are different. A study (23) showed that age, positive lymph node staging, and complete resection were important prognostic factors for long-term survival after lung cancer surgery. Some studies (24, 25) also showed that age was not a risk factor for recurrence. Our study showed that age was associated with postoperative recurrence in NSCLC patients, older patients were more likely to relapse, and the risk of recurrence in patients over 60 years was 2.135 times higher than under 60 years.

In the absence of distant metastasis, whether metastasis involves the ipsilateral mediastinal lymph node (N2) is the most determinant of treatment and survival in patients with lung cancer. Stage N2 is considered to be a more advanced stage of lung cancer, and patients with that stage are more likely to recur than patients with stage N1 (26). In a study of 39,731 patients based on SEER database, Dai et al. (27) found that LNM was an independent factor of prognosis in NSCLC patients. Our study showed that patients in the LNM group were more likely to have recurrence after surgery, and the probability of recurrence was 3.681 times higher than that in the negative group, and the sensitivity for predicting recurrence was the highest among all predictive factors, which was consistent with the conclusions of previous studies.

Chansky et al. (28) studied 9,137 surgically treated NSCLC patients and found that stage was the most important prognostic factor, while cell type was less important. Wang et al. (29) reviewed the clinicopathological characteristics and prognostic factors of 33,919 lung cancer patients and found that TNM stage, gender, age, tumor site, cell type, and differentiation were all independent factors affecting prognosis. Our results found no statistically significant difference in PFS between adenocarcinoma and non-adenocarcinoma patients. Univariate analysis and chi-square test showed that TNM stage was associated with postoperative recurrence, but it was not included in the multivariate analysis. Further analysis revealed that stage III patients are more prone to experience recurrence compared to stage I and II patients. However, the difference in survival between stage II and stage III was not statistically significant. This may be due to the small number of stage II (18 cases) and stage III (31 cases), which can be further analyzed and verified by large sample data in the future.

Melloni et al. (9) found that SUVindex, MTV, and TLG were significantly associated with risk of recurrence, but only TLG seemed to be the most accurate prognostic factor for postoperative recurrence of NSCLC. Laura et al. (30) has found that the diagnostic accuracy of PET/CT for solitary pulmonary nodules (SPNs) is improved by using the ratio between SUVmax in SPN and SUVmean in mediastinal blood pool and between SUVmax in SPN and SUVmean in the liver. In our study, the results of univariate analysis were consistent with it, but only SUVindex could predict postoperative recurrence in multivariate analysis, which may be related to the different cases included in the two studies. Melloni’s study only included stage I patients, while our study included patients who underwent radical resection from stage I to stage III.

Our study also found that there was no statistically significant difference in PFS between patients with adjuvant therapy and without adjuvant therapy.

The heterogeneity and aggressiveness of tumor increased with increasing tumor size (31); for this reason, we also analyzed the effect of GTV and Dmax, which represents the anatomical size of tumor, on PFS. Although the univariate analysis showed that tumor GTV (26.1 cm^3^) and maximum diameter (41.5 mm) were associated with PFS, the results of multivariate analysis were not statistically significant. This also indicates that the larger the tumor, the worse the prognosis, and also suggests that the role of 3D quantitative technique in predicting postoperative recurrence of NSCLC is not particularly prominent.

Although some studies have shown that serum albumin and lymphocyte count have some value in assessing the prognosis of NSCLC (32, 33). We analyzed the results of preoperative serum albumin and lymphocyte in all patients and found that serum albumin and lymphocyte levels were not associated with postoperative recurrence. This may be due to the inclusion of different cases.

There are some limitations in the present study. First, this study is a single-center retrospective study, which may have selective bias and needs to be verified by large sample, multi-center, and prospective studies. Second, only stage I–III NSCLC patients who underwent radical resection and standardized postoperative therapy were included in this study, and patients with more treatment modalities were planned in the next step with lateral and longitudinal prognosis analysis.

In conclusion, 18F-FDG PET/CT combined with 3D quantitative techniques, and clinicopathological features were helpful in predicting PFS in NSCLC patients, but only age, SUVindex, and stage were independent predictors.

## Data Availability

The raw data supporting the conclusions of this article will be made available by the authors, without undue reservation.
